# Distinct gibberellin functions during and after grapevine bud dormancy release

**DOI:** 10.1093/jxb/ery022

**Published:** 2018-01-27

**Authors:** Chuanlin Zheng, Atiako Kwame Acheampong, Zhaowan Shi, Tamar Halaly, Yuji Kamiya, Ron Ophir, David W Galbraith, Etti Or

**Affiliations:** 1 Institute of Plant Sciences, Department of Fruit Tree Sciences, Agricultural Research Organization, Volcani Center, Rishon LeZion, Israel; 2 College of Life Sciences, South China Agricultural University, Guangzhou, China; 3 RIKEN Plant Science Center, Yokohama, Kanagawa, Japan; 4 School of Plant Sciences and Bio5 Institute, University of Arizona, Tucson, AZ, USA

**Keywords:** Bud, dormancy, GA 2-oxidase, GA 3-oxidase, GA 20-oxidase, gibberellin, grapevine, hydrogen cyanamide

## Abstract

The molecular mechanism regulating dormancy release in grapevine buds is as yet unclear. It has been hypothesized that (i) abscisic acid (ABA) represses bud–meristem activity; (ii) perturbation of respiration induces an interplay between ethylene and ABA metabolism, which leads to removal of repression; and (iii) gibberellin (GA)-mediated growth is resumed. The first two hypothesis have been formally supported. The current study examines the third hypothesis regarding the potential involvement of GA in dormancy release. We found that during natural dormancy induction, levels of *VvGA3ox*, *VvGA20ox*, and *VvGASA2* transcripts and of GA_1_ were decreased. However, during dormancy release, expression of these genes was enhanced, accompanied by decreased expression of the bud-expressed GA-deactivating *VvGA2ox*. Despite indications for its positive role during natural dormancy release, GA application had inhibitory effects on bud break. Hydrogen cyanamide up-regulated *VvGA2ox* and down-regulated *VvGA3ox* and *VvGA20ox* expression, reduced GA_1_ levels, and partially rescued the negative effect of GA. GA had an inhibitory effect only when applied simultaneously with bud-forcing initiation. Given these results, we hypothesize that during initial activation of the dormant bud meristem, the level of GA must be restricted, but after meristem activation an increase in its level serves to enhance primordia regrowth.

## Teaser

Gibberellin has an inhibitory effect during initial activation of dormant grapevine buds, and at this stage its level is down-regulated. At a later stage, gibberellin level increases and enhances bud regrowth.

## Introduction

The molecular mechanism regulating dormancy release in grapevine buds in response to either natural or artificial stimuli is as yet unclear, and this limits its manipulation to optimize grape production (Or, 2009). We have previously proposed a working model that describes the biochemical pathways that are involved in artificially induced bud dormancy release (Ophir *et al.*, 2009). According to this model, perturbation of cytochrome pathway activity in the mitochondria leads to respiratory stress. In response to this energy crisis, anaerobic respiration is up-regulated, which mimics hypoxia and thereby affects interplay between ethylene and abscisic acid (ABA) metabolism, which in turn leads to gibberellin (GA)-mediated growth resumption (Ophir *et al.*, 2009). The results of various analyses have supported the predictive power of the model regarding the involvement of respiratory stress, hypoxia, ethylene, and ABA (Zheng *et al.*, 2015, and references within; Vergara *et al.*, 2017). The potential involvement of GA in the cascade that leads to grapevine bud dormancy release, which has not yet been addressed, is the theme of the current study.

GA_12_ biosynthesis from geranylgeranyl diphosphate (GGDP) proceeds in several steps, respectively regulated by *ent*-copalyl diphosphate synthase (CPS), *ent*-kaurene synthase (KS), *ent*-kaurene oxidase (KO), and *ent*-kaurenoic acid oxidase (KAO). Regulation of GA biosynthesis occurs mainly at the later stages of the pathway, for which the relevant enzymes are members of the GA 20-oxidase (GA20ox) and GA 3-oxidase (GA3ox) families that convert GA_12_ to GA_1_ and GA_4_, the major bioactive GAs in plants. GA 2-oxidase (GA2ox), on the other hand, catalyzes GA_1_ and GA_4_ deactivation (Yamaguchi, 2008, and references within). Various factors modulate the levels of GA and, among those, is GA itself, which regulates its own metabolism by down-regulating *GA20ox* and *GA3ox*, and up-regulating *GA2ox* expression (Yamaguchi, 2008, and references within; Middleton *et al.*, 2012).

Bioactive GA activates its response pathway by binding to its nuclear receptors, GA INSENSITIVE DWARF1s (GID1s). This complex then targets DELLAs, the major negative regulators of the GA response, for degradation via binding with SLEEPY1 (SLY1), a GA-specific F-box protein (Hirano *et al.*, 2008; Sun, 2010, 2011; Hauvermale *et al.*, 2012).

Bioactive GAs control many plant developmental processes (Richards *et al.*, 2001), including seed germination (Ogawa *et al.*, 2003) and stem elongation (Peng *et al.*, 1997). It is well established that they promote cell expansion (Dill and Sun, 2001; van den Heuvel *et al.*, 2001; Strader *et al.*, 2004; Ubeda-Tomás *et al.*, 2008; Hauvermale *et al.*, 2012), and recently it has been demonstrated that they also control cell proliferation (Achard *et al.*, 2009; Ubeda-Tomás *et al.*, 2009; Hauvermale *et al.*, 2012; Davière *et al.*, 2014). GAs are also known for their antagonistic relationships with ABA, in regulation of various developmental processes (Weiss and Ori, 2007), including seed dormancy/germination, the most advanced model in the field of dormancy. In this case, GA enhances seed germination across a wide range of plant species, whereas ABA enhances dormancy induction and regulates its maintenance (Seo *et al.*, 2006). This GA–ABA antagonistic relationship is depicted by the following. (i) Increased expression of genes coding for enzymes which catalyze GA catabolism and ABA synthesis (*GA2ox2* and *NCED6*) as seed dormancy becomes deeper. The expression of these genes decreases when dormancy declines, in parallel with increased expression of genes coding for enzymes that catalyze GA synthesis (*GA3ox1*) and ABA catabolism (*CYP707A2*) in Arabidopsis seeds (Footitt *et al.*, 2011). (ii) Bioactive GA-induced germination of lettuce seeds is accompanied by decreased levels of the *LsNCED4* transcript and ABA (Gonai *et al.*, 2004; Sawada *et al.*, 2008). (iii) Increased expression of *AtGA3ox* and *AtGA20ox* and decreased expression of *AtGA2ox6* is evident in the developing seeds of an Arabidopsis mutant having impaired ABA biosynthesis (*aba2-2*), as compared with wild-type plants (Seo *et al.*, 2006). It has therefore been suggested that GA is essential for seed germination, and that ABA plays an important role in depleting bioactive GA levels during dormancy by regulation of expression of GA metabolism genes.

A role for GA during seed germination is further supported by the necessity for functional GA signaling during germination. Accordingly, mutations in *SLY1* lead to increased Arabidopsis seed dormancy, and a triple knockout of *GID1A*, *GID1B*, and *GID1C* results in germination failure (Steber *et al.*, 1998; Griffiths *et al.*, 2006).

Both negative and positive effects of GA were reported on outgrowth of paradormant buds which are carried on actively growing shoots and are under the control of apical dominance. In rice, GA deficiency driven by increased expression of *GA2ox* or decreased expression of *GA3ox* resulted in increased outgrowth of tillers (Dai *et al.*, 2007; Lo *et al.*, 2008). Similarly, overexpression of *AtGA2ox* in bahiagrass resulted in reduced GA_1_ and enhanced outgrowth of axillary buds (Agharkar *et al.*, 2007). In hybrid aspen, decreased bioactive GA levels in the apex and young tissues (mediated by *GA2ox* overexpression) resulted in loss of apical dominance and increased branching of lateral buds. Interestingly, a similar decrease in mature vasculature did not present such an effect (Mauriat *et al.*, 2011). On the other hand, indications for positive regulation of paradormant bud outgrowth by GA was also recorded. Light-induced branching of *Rosa* sp. laterals was accompanied by increased expression of *GA20ox* and *GA3ox*, and decreased *GA2ox*, but GA could not rescue bud burst in the dark (Choubane *et al.*, 2012). The Arabidopsis *phyb* mutant presented decreased branching and increased *GA2ox* (Su *et al.*, 2011). GA biosynthesis inhibitors inhibited cytokinin (CK)-mediated outgrowth of axillary buds of *Jatropha curcas*, whereas GA promoted it, and also down-regulated expression of inhibitors of bud outgrowth from the BRC family (Ni *et al.*, 2015). GA has also been reported to induce lateral bud outgrowth in papaya (Ni *et al.*, 2015). Finally, in paradormant buds of hybrid aspen, the level of the *GA3ox2* transcript was low during maturation, and was induced in response to decapitation (Rinne *et al.*, 2016).

Several studies have reported an increased level of endogenous GA, or an up-regulation of GA biosynthesis genes, following exposure of perennial endodormant buds to chilling, or fluctuations in the levels of GA during the natural bud endodormancy cycle. In sweet cherry, the endogenous ABA/GA_3_ ratio in flower buds increased during natural dormancy induction and decreased during dormancy release (Duan *et al.*, 2004). An increased level of GA following exposure to chilling was reported in dormant buds of peach (Frisby and Seeley, 1993). In agreement, low temperature treatments induced the expression of members of the *GA20ox* (Karlberg *et al.*, 2010) and *GA3ox* (Rinne *et al.*, 2011) gene families in hybrid aspen dormant buds, while down-regulating expression of paralogs of *GA2ox* (Karlberg *et al.*, 2010).

Application of GA had contrasting effects (inhibition or induction) on bud break during and after winter dormancy of temperate perennial buds. It was reported that application of GA_3_ enhanced bursting of dormant Elberta peach buds (Donoho and Walker, 1957), and GA_4_ application enhanced dormancy release of Japanese apricot flower buds (Zhuang *et al.*, 2013). In *Populus*, treatment with 1–100 µM GA_4_ induced bud burst, but treatment with GA_3_ resulted in bud abscission (100 µM GA_3_) and protrusion of embryonic leaves (0.1–10 µM GA_3_) (Rinne *et al.*, 2011). In kiwi fruit, application of GA_3_ prior to chilling enhanced dormancy, while application after exposure to chilling promoted bud break (Lionakis and Schwabe, 1984). GA_3_ application also delayed dormancy release of grape and persimmon buds (Weaver, 1959; Iwasaki, 1980; Kang *et al.*, 1998). In agreement with the above, it was formerly proposed that the observed increase in endogenous GA may be a result, rather than a cause, of bud dormancy removal, and might regulate bud expansion during regrowth (Saure, 1985; Lang, 1994).

In the current study, we describe a detailed examination of the potential involvement of GA in the artificially and naturally stimulated cascades that lead to grapevine bud dormancy release.

## Materials and methods

### Plant material

Analyses of dormancy status and of the effects of GA, hydrogen cyanamide (HC), HC–GA, hypoxia, heat shock (HS), and chilling treatments on dormancy release were conducted using mature buds collected from cordon-trained grapevines (*Vitis vinifera* cv. Early sweet, grafted on 140 Ruggeri) in a commercial vineyard, planted on calcareous rendzina soil at Gilgal located in the Jordan Valley, Israel. The region is characterized by warm winters (20/10 °C mean daily maximum/minimum in January; 150 mm rainfall) and hot summers (38/23 °C in June). Vine spacing was 1.5 m within rows and 3 m between rows, and the vines were trained to a Y-shaped, open-canopy gable system. Drip irrigation with drippers spaced by 50 cm was computer controlled. The irrigation control unit was set to 40% of reference evapotranspiration (ET_o_) during bud break, and the amount of water applied was gradually increased to 100% of ET_o_ at harvest. After harvest, it was gradually reduced to 40% of ET_o_. The meteorological data used for calculating ET_o_ were obtained from a weather station located at Gilgal. Irrigation frequencies ranged from once a week (during bud break) to three times a week from veraison until harvest. NPK liquid fertilizer (Tuv, ICL, Haifa, Israel) was applied through the irrigation system at a rate of 1–1000 liters of water, at a ratio of 6–3–9 from bud break until veraison, and 0–0–15 from veraison until harvest. Vines were pruned to three-node spurs, and the detached canes, each carrying nine buds (in positions 4–12), were transferred to the lab. Field experiments were conducted in a commercial vineyard of the same cultivar at Argaman, in the Jordan Valley.

### Natural dormancy curve

To create a dormancy curve that describes changes in dormancy status throughout the natural dormancy cycle, bud break was monitored at 1 week intervals from November to the January of the following year, as described in Zheng *et al.* (2015). Single-node cuttings prepared from canes were randomly mixed, and nine groups of 10 cuttings were placed in vases containing tap water. The vases were placed in a growth chamber and the cuttings forced at 22 °C under a 14/10 h light/dark regime. Bud break was defined as the stage at which green tissue became visible underneath the bud scales (Or, *et al.*, 2002). Bud break was typically monitored at days 7, 11, 14, 18, and 21 following completion of an initial adjustment period of 48 h. Bud break percentages at 21 d were used to describe the seasonal changes in dormancy status of the bud population in the vineyard (Zheng *et al.*, 2015).

For gene expression and hormone analyses, three groups of 100 buds were randomly sampled each week from the pool of single-node cuttings, on the day of arrival from the vineyard. These three bud pools were frozen in liquid nitrogen and stored at –80 °C.

### Analyses of the effect of chemical and physical treatments on bud break

To test the effect of HC (3% Dormex^®^, v/v), HS (immersed in 50 °C water for 1 h), and hypoxia (forced at 1% O_2_ up to 48 h) on bud dormancy release, cuttings were treated as described in Zheng *et al.* (2015). After treatment, bud break was monitored under the forcing conditions described previously. For gene expression and hormone analyses, buds were sampled at 12, 24, 48, and 96 h for control, HC, and HS treatments, frozen in liquid nitrogen, and stored at –80 °C. Hypoxia treatments and the corresponding controls were carried out as previously described (Zheng *et al.*, 2015), and sampled at 24 h and 48 h after sealing the jars.

### Analyses of the effect of GA on bud break

To test the effect of GA on bud dormancy release, cuttings were sprayed with GA_3_ (stock solution: 33 g l^–1^ GA_3_, Pro-Gibb; Abbott Laboratories, Chicago, IL, USA) and placed in vases with tap water under the forcing conditions described previously. For schematic details of all the GA treatments, see Supplementary Table S1 at *JXB* online. Initially, two series of GA_3_ concentrations were used in two different experiments: 1.25, 2.5, 5, 10, 20, and 40 ppm (where 1 ppm GA_3_ equals 2.887 µM) for the first experiment, and 0.001, 0.01, 0.1, and 1 ppm for the second experiment. Additional experiments similarly tested the effect of application of a mix of GA_4_ and GA_7_ [10 ppm; GA_4 + 7_ (2:1), Duchefa, Haarlem, The Netherlands].

All GA solutions were formulated in 0.02% (v/v) Triton X-100. The control was treated similarly with 0.02% Triton X-100 solution. For the combined HC–GA treatments, cuttings were treated with a mixture of HC and 1, 5, or 10 ppm GA. To test the effect of GA on HC-triggered bud dormancy release further, GA (10 ppm) was applied at 0, 2, 6, and 10 d after HC treatment. Bud break was monitored after treatments under the forcing conditions previously described. To test the effect of GA on dormancy release of whole vines under vineyard conditions, cv. Early Sweet vines in a commercial vineyard, located in Argaman, Jordan Valley, were pruned to three-node spurs on 14 January 2014 and sprayed with 0.02% Triton X-100 without (control) or with 10 ppm GA_3_ (GA) to runoff (~1 liter per vine), using a 15 liter knapsack sprayer (SOLO^®^, VA, USA). Each treatment consisted of four blocks of three vines, in a randomized complete block design. A day after pruning, bud break was monitored for each vine in each block. The total number of buds was determined on 14 January, and the numbers of bursting buds were counted on 24 February, 4 March, and 11 March.

To test the effect of GA on pre-chilled buds, detached canes collected on 22 November 2015 were sprayed with 0.1% (w/v) fungicide (Switch^®^ 62.5 WG; Syngenta), packed with plastic film, and stored at 4 °C for 1000 h. Canes were then used to prepare three groups of single-node cuttings, each with nine subgroups of 10 cuttings. One group was immediately treated with 1 ppm GA_3_. Two additional groups were treated with 0.02% Triton X-100: one was used as control and the other was treated, 7 d later, with 1 ppm GA_3_, when visible green tissue was detected on the first bud in the entire group. Forcing and bud break monitoring procedures were as described above.

To test the effect of GA on bud break of ecodormant buds, canes were collected from the vineyard after endodormancy release (18 February 2016), and groups of were cuttings prepared and treated as described for pre-chilled buds, except for the late GA treatment which was carried out 3 d after treatment with Triton X-100.

To test the effect of GA_3_ (10 ppm) on gene expression, the procedure described for analyses of the effect of chemical treatments was employed.

### RNA extraction and quantitative real-time PCR (qRT-PCR) analyses

Total RNA was extracted from 20 frozen buds using cetyltrimethylammonium bromide (CTAB) as previously described (Acheampong *et al.*, 2010). RNA was treated with RQ DNase (Promega, Madison, WI, USA) according to the manufacturer’s instructions, and dissolved in 40 μl of DNase/RNase-free water. cDNA was synthesized from total RNA using M-MLV Reverse Transcriptase (Promega) as detailed in the instruction manual.

Relative transcript levels of bud-expressed paralogs of *VvGA2ox*, *VvGA3ox*, and *VvGA20ox* gene families throughout the natural dormancy season and in response to HC were determined using the 96X96 Dynamic array Integrated Fluidic Circuits (96 IFCs) on the Biomark platform (Fluidigm, San Francisco, CA, USA) according to the manufacturer’s protocol. Transcript levels in buds exposed to hypoxia, HS, or GA treatments, and the relevant controls, were quantified by qRT-PCR with ABsolute Blue QPCR SYBR Green Low ROX Mix (Thermo Fisher Scientific, Waltham, MA, USA) on a Corbett Rotor-Gene 6000 (Qiagen, Hilden, Germany). *VvActin* and *VvGAPDH* primers, characterized and optimized by Reid *et al.* (2006), were used for normalization, while *VvGA2ox*, *VvGA3ox*, *VvGA20ox*, and *VvGASA2* primers were previously optimized by Giacomelli *et al.* (2013) and Acheampong *et al.* (2017). All the primers are presented in Supplementary Table S2.

### Quantitation of endogenous GA_1_ and GA_4_

Triplicate samples of 10 frozen buds for each biological replicate were homogenized in liquid nitrogen, and 0.5 g of the homogenized powder was used for hormone extraction and GA quantitation, as previously described (Acheampong *et al.*, 2015). Endogenous GA levels were calculated from the ratios of the peak areas of these endogenous compounds to internal standards.

### Statistical analyses

Statistical analyses were performed on a JMP 12.0.1 (SAS Institute, Cary, NC, USA) by one-way ANOVA with Student’s *t*-test (*P<0.05), or with Tukey’s HSD with a *P*-value <0.05.

## Results

### Profiling of *VvGA3ox*, *VvGA20ox*, and *VvGA2ox* transcript levels in grapevine buds during the natural dormancy cycle

Five putative *VvGA20ox* genes, three *VvGA3ox* genes, and nine *VvGA2ox* genes were predicted, using Arabidopsis amino acid sequences as reference (Jung *et al.*, 2014). The number of *VvGA20ox* and *VvGA2ox* genes was adjusted to six and eight, respectively, by parallel comparisons with *Glycine max* and *Oryza sativa*, and activity tests (Giacomelli *et al.*, 2013). Of those, two *VvGA20ox* genes (*VvGA20ox3* and *VvGA20ox6*) and two *VvGA3ox* genes (*VvGA3ox1* and *VvGA3ox2*) are expressed in mature woody grapevine buds. Of the *VvGA2ox* gene family members with previously confirmed GA deactivation activity, *VvGA2ox3* is the paralog that is most highly expressed in the bud, while *VvGA2ox4* is expressed at a lower level (Fasoli *et al.*, 2012; Giacomelli *et al.*, 2013; Khalil-Ur-Rehman *et al.*, 2017). All these genes, except for *VvGA3ox1*, are under GA feedback regulation (Supplementary Fig. S1) as expected (see the Introduction).

**Fig. 1.**
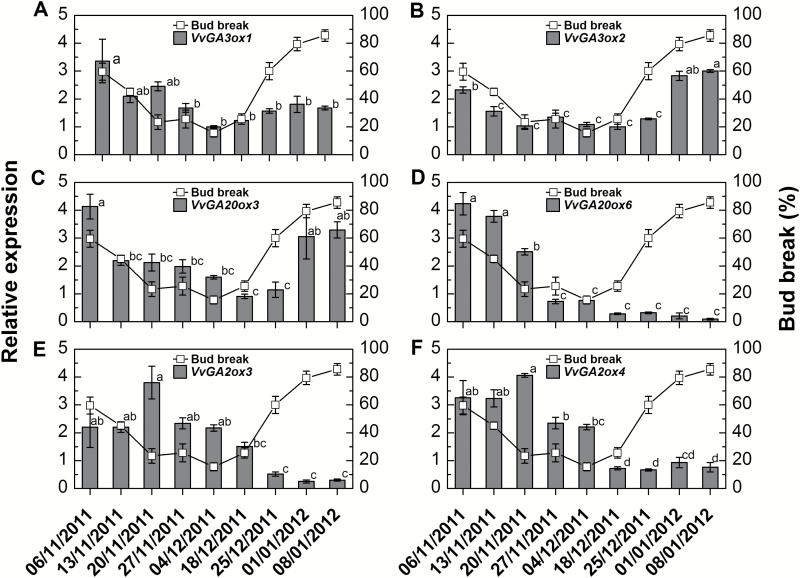
Expression profile of genes encoding central components of GA metabolism throughout the dormancy cycle. Vines (*Vitis vinifera* cv. Early Sweet) from a vineyard at Gilgal, located in the Jordan Valley, were pruned to three-node spurs. The detached canes were sampled weekly throughout the dormancy cycle, cut into single-node cuttings, randomly mixed, and nine groups of 10 cuttings were prepared and placed in water-filled vases. The vases were placed in a growth chamber, at 22 °C under a 14/10 h light/dark regime. The bud break percentages at 21 d are shown (as line) in each panel. Values are averages of nine groups of replications, consisting of 10 buds each ±SE. Total RNA was extracted from buds sampled weekly, upon arrival from the vineyard, and frozen. Relative transcript levels were determined for *VvGA3ox1* (A), *VvGA3ox2* (B), *VvGA20ox3* (C), *VvGA20ox6* (D), *VvGA2ox3* (E), and *VvGA2ox4* (F), using qRT-PCR (see the Materials and methods) and normalized against *VvActin* and *VvGAPDH*. Values of qRT-PCR represent the mean ±SE of three biological replications, each with two technical repeats. Data points with different letters indicate significantly different values (*P*<0.05) according to Tukey’s HSD test.

To assess the potential involvement of GA metabolism in the execution of the natural dormancy cycle, the transcript levels of the bud-expressed *VvGA3ox*, *VvGA20ox*, and *VvGA2ox* paralogs were quantified in buds collected from the beginning of November to the beginning of January by qRT-PCR. Transcript levels for all four GA biosynthesis genes gradually decreased during the dormancy induction period (Fig. 1A–D), and were lowest on 4 December (*VvGA3ox1*) or 18 December (*VvGA3ox2*, *VvGA20ox3*, and *VvGA20ox6*), two time points which lie within the period of dormancy maintenance. Transcript levels of *VvGA3ox2* and *VvGA20ox3* sharply increased during dormancy release, whereas a more gradual increase, which was not significant, was recorded for *VvGA3ox1*. Interestingly, *VvGA20ox6* expression, which presented a profile similar to that described above during dormancy induction and maintenance, did not increase during dormancy release. It may be worth noting that the functionality of this gene as a *GA20ox* has not yet been validated, as it could not be amplified from Pinot Noir (Giacomelli *et al.*, 2013).

The transcript levels for *VvGA2ox3* were rather stable up to the deepest stage of dormancy/dormancy maintenance (represented by 4–18 December), with one exception on 20 November—the transition point to deep dormancy (Fig. 1E). Subsequently, transcript levels gradually decreased from 18 December to 8 January, with a corresponding increase in bud break ability. The expression profile of *VvGA2ox4* (Fig. 1F) was similar to that of *VvGA2ox3* (Fig. 1E), including one point of non-significant up-regulation noted for 20 November, suggesting that the mode of regulation was similar throughout the dormancy cycle.

### Levels of bioactive GAs in grapevine buds during the dormancy cycle

**Fig. 2.**
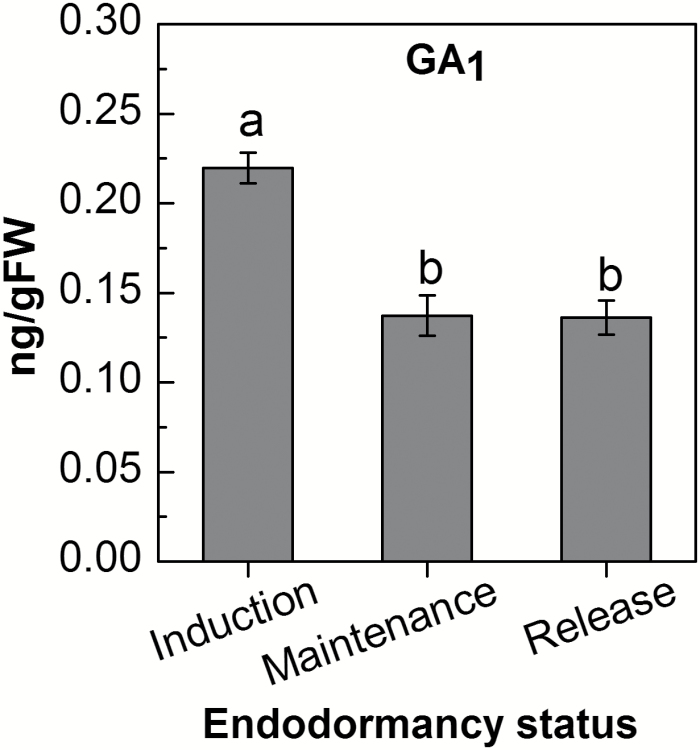
Quantities of endogenous GA_1_ in grapevine buds throughout the dormancy cycle. Buds were sampled weekly from the vineyard and were frozen immediately as described in Fig. 1. Buds were homogenized, and GA_1_ was quantified by LC–MS/MS (Acheampong *et al.*, 2015). ^2^H-labeled GA_1_ was spiked as internal standard. The levels of GA_1_ were calculated from the peak area ratios of the endogenous GA_1_ to its ^2^H-labeled internal standard. Three biological replications (10 buds per replicate) were analyzed for each time point, and means from three time points during endodormancy induction, maintenance, and release (1–13 November, 20 November–18 December, and 25 December–8 January, respectively) served to calculate GA_1_ levels. Data points with different letters indicate significantly different values (*P*<0.05) according to Tukey’s HSD test.

Levels of GA_1_ and GA_4_, the predominant bioactive GAs in grapevine (Giacomelli *et al.*, 2013; Acheampong *et al.*, 2015), were determined in grape buds which were sampled from a commercial vineyard throughout the natural dormancy cycle. Whereas GA_4_ was undetectable in any of the endodormancy phases, the levels of GA_1_ were relatively high during dormancy induction, and decreased ~40% during dormancy maintenance. No further changes were detected during endodormancy release in the ecodormant buds sampled from the vineyard for this analysis (Fig. 2).

### Profiling of the transcript level of the GA-responsive gene *VvGASA2* during the natural dormancy cycle

**Fig. 3.**
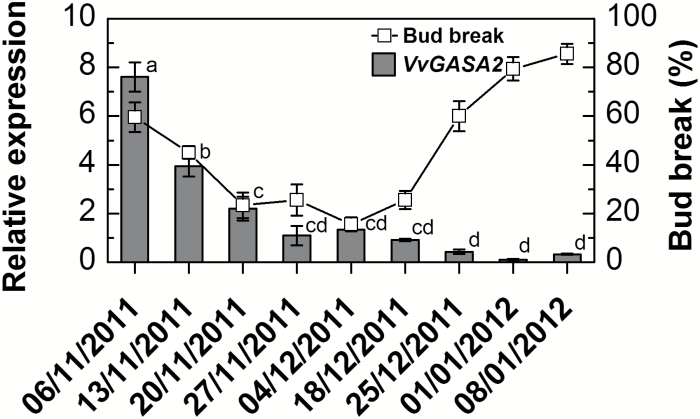
Expression profile of a gene encoding a grapevine *AtGASA4* ortholog, *VvGASA2*, throughout the dormancy cycle. All the details are as in Fig. 1.

To address further GA responses during the natural dormancy cycle, we followed potential changes in the expression of *VvGASA2*, a grapevine homolog of the GA-responsive *GAST1 PROTEIN HOMOLOG 4* (*AtGASA4*) (Acheampong *et al.*, 2017). The GA-responsive nature of *VvGASA2* expression in grapevine buds was initially confirmed, as shown by significant increases of its transcript level following GA_3_ application (1.9-and 1.6-fold increase after 48 h and 96 h from treatment, compared with control, Supplementary Fig. S1). Profiling of its transcript level throughout the natural dormancy cycle (Fig. 3) revealed the highest levels during dormancy induction, with a gradual decrease towards dormancy maintenance. No further changes were detected during endodormancy release in the ecodormant buds.

### Effect of exogenous GA_3_ application on dormancy release of grapevine buds

**Fig. 4.**
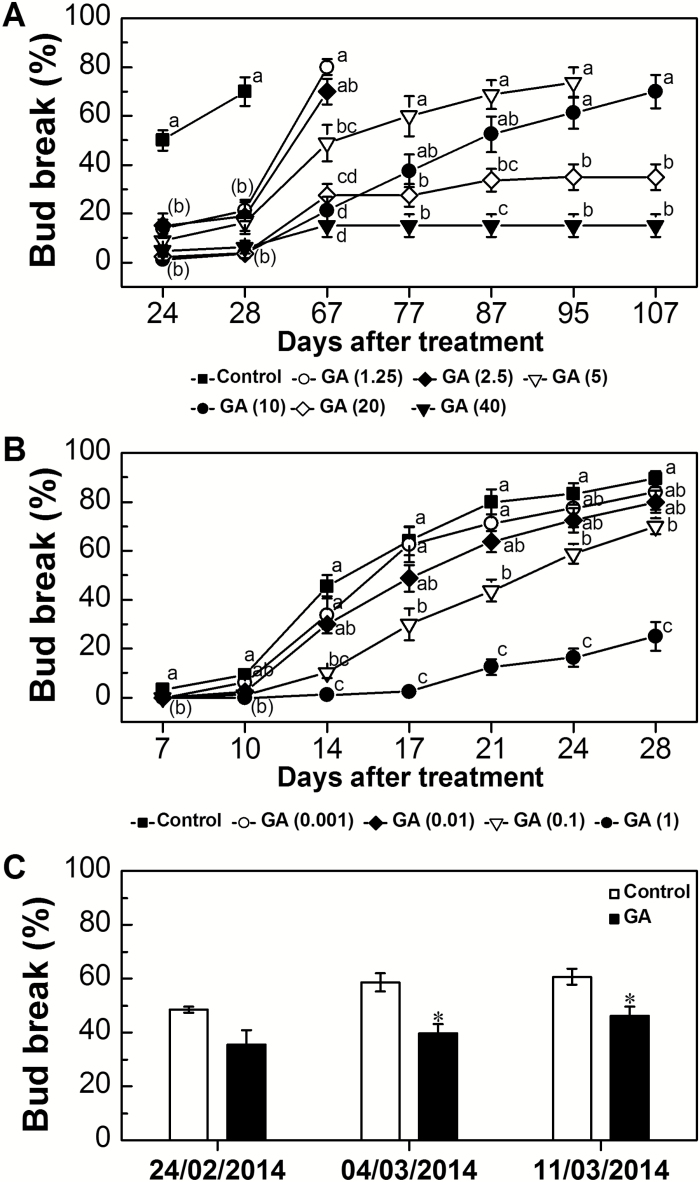
The effect of GA_3_ application on bud break of single-node cuttings and whole vines. (A) Cuttings were sprayed with 1.25, 2.5, 5, 10, 20, and 40 ppm GA_3_ (where 1 ppm GA_3_ equals 2.887 µM) formulated with 0.02% Triton X-100, placed in vases, and monitored for 107 d. Triton X-100 (0.02%)-treated buds served as control. Each treatment was monitored until a bud break percentage similar to that of control at 28 d (of ~70%) was attained or the buds became stunted. For additional details, see Fig. 1. (B) Cuttings were sprayed with 0.001, 0.01, 0.1, and 1 ppm GA_3_ and monitored for 28 d. For additional details, see Fig. 1. (C) Vines were pruned to three-node spurs on 14 January 2014. GA (10 ppm GA_3_ with 0.02% Triton X-100 solution) and control (0.02% Triton X-100 solution) treatments were conducted on 15 January. Four blocks of three vines were used, and bud break was monitored separately for each vine in each block. The total number of buds was recorded and the number of bursting buds was counted on 24 February, 4 March, and 11 March. Bars represent the average bud break of the 12 grapevines in the four blocks for each treatment ±SE. Statistical tests indicate differences between treatments at each time point. Data points with different letters indicate significantly different values (*P*<0.05) according to Tukey’s HSD test. Asterisks between treatments indicate significantly differences according to Student’s *t*-test (*P<0.05).

To test the hypothesis that GA enhances dormancy release of grapevine buds, the response of dormant buds to GA_3_ was compared with that of Triton X-100. Contrary to the initial hypothesis, application of GA to dormant buds significantly inhibited bud break (Fig. 4). The results (Fig. 4A) suggested that both time- and concentration-dependent effects exist, since: (i) up to 28 d from GA application, all analyzed concentrations of GA significantly inhibited bud break as compared with the control, which presented ~70% bud break at 28 d; and (ii) monitoring over an extended period of time allowed observation of recovery, yet the degree and the rate of recovery were concentration dependent. Low GA concentrations (1.25 ppm and 2.5 ppm) allowed recovery from the inhibitory effect after 67 d, leading to 80% and 70% bud break, respectively. Application of 5 ppm and 10 ppm GA resulted in a slower and lower recovery rate, leading to 75% and 70% bud break only after 95 d and 107 d, respectively. Higher concentrations of GA (20 ppm and 40 ppm) severely inhibited bud break, with only a slight recovery between 28 d and 67 d (reaching ~35% and 15% bud break, respectively), and complete stagnation beyond 87 d and 67 d, respectively.

A second set of experiments tested the effect of GA application at lower concentrations (0.001–1 ppm GA_3_) for a shorter (28 d) monitoring period (Fig. 4B). Low GA_3_ (0.001 ppm) had no inhibitory effect on bud break. A non-significant inhibitory effect was recorded when buds were treated with 0.01 ppm GA_3_, and the inhibitory effect gradually and significantly increased with increased GA_3_ concentrations. At a concentration of 1 ppm, severe inhibition was recorded across the entire period of analysis.

A third experiment, which tested the effect of application of a mix of GA_4_ and GA_7_ (10 ppm, ratio of 2:1) for 25 d (Supplementary Fig. S2) also resulted in significant inhibition of bud break, as compared with the control.

To test whether the negative effect of GA on bud break is also observed under natural conditions, a field experiment was conducted. Application of GA (10 ppm) on 15 January resulted in a significant decrease of 19% and 14% in bud break on 4 March and 11 March, respectively, as compared with Triton X-100-treated control buds (Fig. 4C).

### Effect of HC on the bud break inhibition induced by exogenous GA, and the endogenous GA content of grapevine buds

**Fig. 5.**
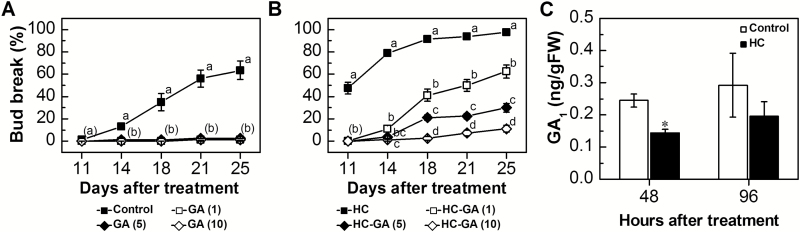
The effect of GA_3_ on HC-triggered bud break. (A and B) GA buds received a single treatment of 1, 5, or 10 ppm GA_3_. HC–GA buds were treated with the above GA concentrations concomitantly with HC (3% Dormex^®^). All solutions were formulated in 0.02% Triton X-100, which served as control treatment. All other details are as in Fig. 1. Bud break was recorded 11, 14, 18, 21, and 25 d after treatment. Values represent average bud break of nine groups of 10 cuttings ±SE. (C) GA_1_ levels were determined in control and HC-treated buds sampled at 48 h and 96 h after treatment as described in Fig. 2. Bars represent means ±SE of three biological repeats (10 buds per repeat). Data points with different letters indicate significantly different values (*P*<0.05) according to Tukey’s HSD test. Asterisks between treatments indicate significant differences according to Student’s *t*-test (*P<0.05).

The effect of HC on the bud break inhibition induced by treatment with exogenous GA was tested by combined application (HC–GA; Fig. 5B) which was compared with net GA application (GA; Fig. 5A). The results were as follows. (i) Addition of HC in HC–GA treatment (Fig. 5B) rescued the inhibitory effect of 1 ppm GA (Fig. 5A), resulting in bud break that is similar to that of the untreated control (Fig. 5A). However, addition of HC failed to rescue fully the negative effect of higher concentrations of GA (5 ppm and 10 ppm), when compared with GA and control treatments. (ii) GA at 1–10 ppm significantly attenuated the enhancing effect of HC on bud break, and the degree of attenuation was concentration dependent (35, 68, and 86% decreases in bud break at 25 d in response to GA concentrations of 1, 5, and 10 ppm GA in HC–GA treatment, as compared with HC treatment, Fig. 5B).

The levels of the grapevine bioactive GA molecules GA_1_ and GA_4_ were quantified in HC-treated and control buds sampled at 48 h and 96 h after treatment. GA_4_ was undetectable in all treated buds. GA_1_ was detected and its level significantly decreased (41%) at 48 h after HC treatment (Fig. 5C), as compared with control. At 96 h there was no significant difference between HC-treated and control buds.

### Effect of HC treatment on expression of central components of GA metabolism in grapevine buds

It was formerly suggested that: (i) the increased level of GA in both seeds and buds is a result, rather than a cause, of dormancy removal; and (ii) the decrease in ABA levels may be a prerequisite for elevations in GA levels and in GA sensitivity within seeds (see the Introduction). Integration of these assumptions with the results described above provides the speculation that GA might have a negative effect on grape bud dormancy release, but, nonetheless, a positive effect on regrowth after dormancy release. Following this rationale, stimuli of dormancy release might be involved in relieving the GA repression during dormancy release via modification of GA metabolism. To test this assumption, comparative transcript profiling of central regulators of GA biosynthesis and degradation was carried out, using HC-treated and control buds.

**Fig. 6.**
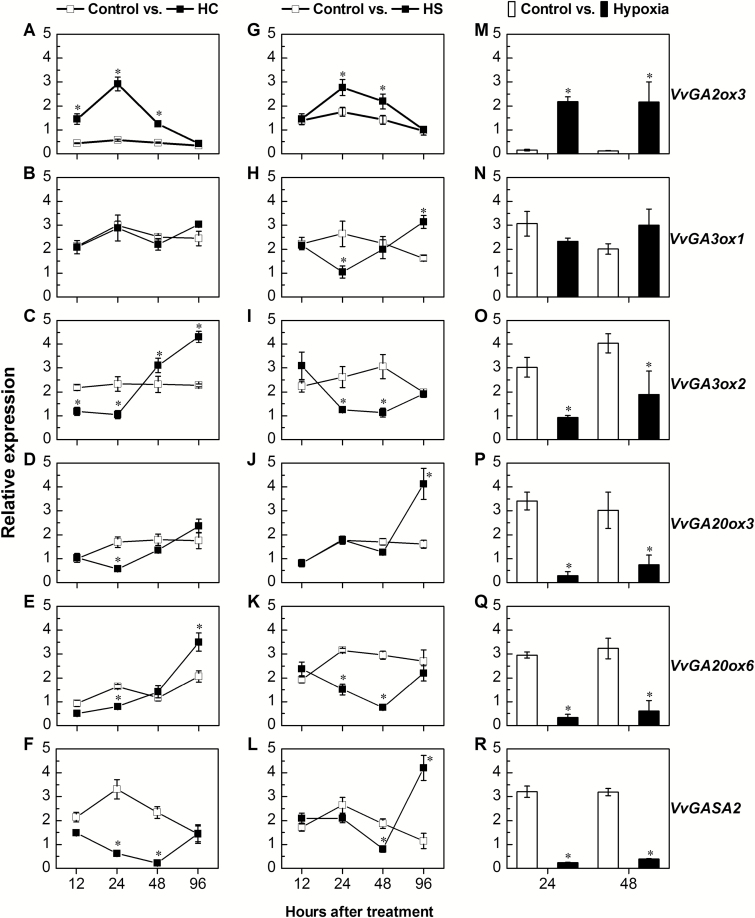
The effect of artificial stimuli of dormancy release on the expression profile of central components of GA metabolism and the GA-responsive *VvGASA2* gene. Total RNA was extracted from control- (0.02% Triton X-100), HC- (3% Dormex^®^ with 0.02% Triton X-100), and HS- (50 °C water for 1 h) treated buds sampled at 12, 24, 48, and 96 h after treatment, and from hypoxia- (1% O_2_) treated buds at 48 h and 96 h after treatment. Relative expression of *VvGA2ox3* (A, G, and M), *VvGA3ox1* (B, H, and N), *VvGA3ox2* (C, I, and O), *VvGA20ox3* (D, J, and P), *VvGA20ox6* (E, K, and Q), and *VvGASA2* (F, L, and R) was determined by qRT-PCR in HC- (A–F), HS- (G–L), and hypoxia- (M–R) treated buds, as described in the Materials and methods, and normalized against *VvActin* and *VvGAPDH*. Values represent the mean expression ±SE of three biological replications, each with two technical repeats. Asterisks between treatments indicate significant differences according to Student’s *t*-test (*P<0.05).

HC treatment significantly up-regulated the expression of the GA deactivation gene *VvGA2ox3* at 12, 24, and 48 h, with a maximum difference of 5.2-fold at 24 h and no difference at 96 h (Fig. 6A). In contrast, the expression of the GA biosynthesis genes *VvGA3ox2*, *VvGA20ox3*, and *VvGA20ox6* was significantly decreased at 24 h after HC application (Fig. 6C–E). The expression levels of *VvGA3ox2* and *VvGA20ox6* were also significantly increased at 96 h after HC application (Fig. 6C, E). *VvGA3ox1* expression was not significantly regulated by HC at any of the time points (Fig. 6B).

Analyses of the effect of other dormancy release stimuli, hypoxia and HS, were carried out similarly (Fig. 6G–K, M–Q). As seen for treatment with HC, both HS and hypoxia significantly up-regulated *VvGA2ox3* expression at 24 h and 48 h after treatment (Fig. 6G, M), but down-regulated *VvGA3ox2* and *VvGA20ox6* expression (Fig. 6I, K, O, Q). *VvGA3ox1*, which was unaffected by HC, was significantly down-regulated by HS at 24 h, and up-regulated at 96 h (Fig. 6H). Similar differences, also non-significant, were detected in *VvGA3ox1* expression in response to hypoxia (Fig. 6N). In the case of *VvGA20ox3*, significant late (96 h) up-regulation was shown in response to HS (Fig. 6J), and significant down-regulation was evident in response to hypoxia at 24 h and 48 h (Fig. 6P).

Interestingly, expression of the GA-responsive *VvGASA2* was significantly decreased in response to HC, HS, and hypoxia at 24–48 h after treatment (Fig. 6F, L, R), in accordance with the decrease in the levels of *VvGA3ox* and *VvGA20ox* transcripts (Fig. 6C–E, H–I, K, O–Q) and GA_1_ (Fig. 5C), and the increase in levels of the *VvGA2ox* transcript described above (Fig. 6A, G, M). Increases in *VvGASA2* expression were recorded between 48 h and 96 h after HC and HS treatments, but this increase was significantly higher than its level in the control only at 96 h after HS treatment.

### The effect of timing of GA application on bud break inhibition capacity

**Fig. 7.**
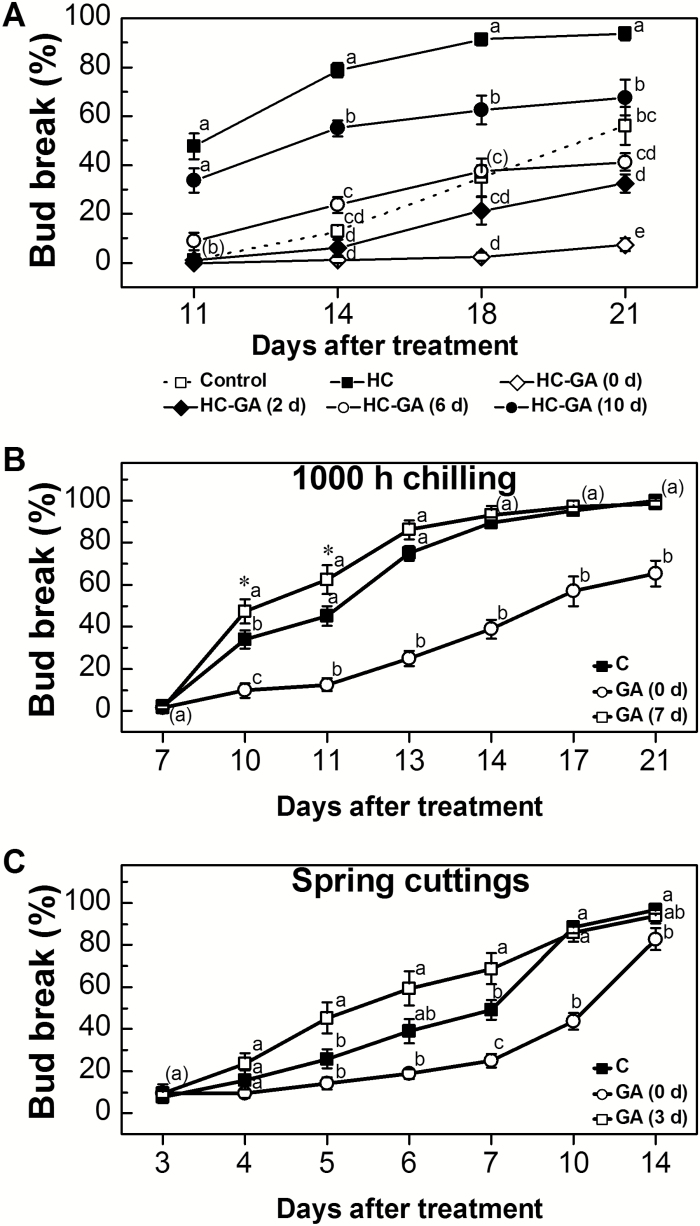
The effect of the timing of application of GA_3_ on bud break. (A) Buds were treated with GA (10 ppm) 0, 2, 6, and 10 d after HC treatment. For additional details, see Figs 4A and 1. (B) Canes were collected on 22 November 2015, pre-chilled at 4 °C for 1000 h, and used to prepare cuttings. A set of nine groups of 10 cuttings were immediately treated with 1 ppm GA_3_, and another two sets (one serving as control treatment, and the other for the delayed 7 d treatment) were treated with 0.02% Triton X-100. Buds were forced for 7 d. At 7 d, one of the two Triton X-100-treated sets was treated with 1 ppm GA_3_. For additional details, see Fig. 1. (C) Canes were collected in the vineyard after endodormancy release (18 February 2016). The experiment was designed and undertaken as described in (B). The delayed GA treatment was carried out after 3 d. Values are averages of the nine groups in each treatment ±SE. Data points with different letters indicate significantly different values (*P*<0.05) according to Tukey’s HSD test. Asterisks between treatments indicate significantly differences according to Student’s *t*-test (*P<0.05).

To understand further the nature of the inhibition exerted by GA, we monitored the effect of timing of GA_3_ application after stimulation of bud break by either artificial or natural means. The inhibitory effect of GA on HC-treated buds at the stage of dormancy release (sampled on 27 December) decreased significantly with increasing time lapsed between HC and GA applications (Fig. 7A). Application of GA concomitantly with HC resulted in 7.5% bud break after 21 d, whereas GA application 2, 6, and 10 d after HC treatment resulted in 32.5, 41.3, and 67.5% bud break, respectively, after 21 d. Similar results were obtained when buds, previously exposed to 1000 h of chilling, were treated with GA (Fig. 7B). Treating pre-chilled buds with GA at the beginning of the forcing process resulted in 24.3, 32.6, 50.0, 50.7, 38.2, and 34.7% inhibition of bud break, at 10, 11, 13, 14, 17, and 21 d, compared with untreated chilled buds. However, application of GA 7 d later enhanced bud break by 13.2, 17.4, and 11.1% at 10, 11, and 13 d, respectively (Fig. 7B). The bud break values of the GA (0 d) treatment were statistically different from those of the untreated chilled buds and the GA- (7 d) treated buds. The GA- (7 d) treated buds exhibited significant differences from the untreated buds only at 10 d (or at 10 d and 11 d, when analyzed separately by Student’s *t*-test). In a third analysis (Fig. 7C), GA was applied to bud populations after the natural completion of the endodormancy cycle (on 28 February). Application of GA at the beginning of the forcing process resulted in significant inhibition of bud break, as compared with the untreated control (24.2% and 44.5% inhibition of bud break at 7 d and 10 d, respectively), whereas application of GA 3 d after forcing had a significant enhancing effect, as compared with the untreated control (19.5% and 19.5% enhancement of bud break at 5 d and 7 d). Significant differences were evident between the two GA treatments at 5, 6, 7, and 10 d (31.3, 40.6, 43.8, and 42.2%, respectively).

## Discussion

### Changes in regulation of GA metabolism during the natural dormancy cycle of grapevine buds

The transcript profiles of GA metabolism genes recorded in the buds throughout the natural dormancy cycle generally agree with the changes in the level of endogenous active GA and the GA-responsive *VvGASA2* transcript, with the assumptions of the proposed cascade that leads to dormancy release (Ophir *et al.*, 2009), and with a recent study in Arabidopsis seeds (Footitt *et al.*, 2011).

The period of dormancy induction was accompanied by a gradual decrease in transcript levels for the bud-expressed *VvGA3ox* and *VvGA20ox* genes (coding for GA biosynthetic enzymes), and their levels were lowest during deepest dormancy. On the other hand, the level of the highest bud-expressed *VvGA2ox* paralog, *VvGA2ox3*, was relatively high during this same period (Fig. 1). Similar behavior was recorded for Arabidopsis seed development under natural conditions, where increased dormancy was accompanied by decreased expression of *GA3ox1* and increased expression of *GA2ox2* (Footitt *et al.*, 2011). The reduced GA biosynthesis capacity and the maintenance of stable GA inactivation ability during dormancy induction of grapevine mature buds correlates with the decrease in level of endogenous active GA_1_ following the period of dormancy induction, and its lower level during dormancy maintenance (Fig. 2). Overall, the presented results support the assumption that the cascade of events leading to natural bud dormancy induction involves a decrease in GA biosynthesis capacity and a maintenance of GA degradation ability, which results in decreased levels of bioactive GA following dormancy induction. Maintenance of low levels of GA may therefore be required during deep dormancy. Such an assumption was recently considered regarding hybrid aspen bud dormancy (Rinne *et al.*, 2016), being justified in terms of the need to restrain growth of the packed embryonic shoot.

During the period of gradual dormancy release, a similar scenario was evident, but with the trends reversed. The level of *VvGA2ox3* transcripts markedly decreased during the transition from deep dormancy (4 December) to the stage of dormancy release (18 December–1 January). In contrast, expression levels of the GA biosynthesis genes generally increased during the period of dormancy release. The most notable increase accompanied acquirement of maximal bud break ability within the bud population under forcing conditions (1–8 January). Similar behavior was recorded for Arabidopsis seeds developing under natural conditions, where the transition from deep dormancy to shallow dormancy was accompanied by increased expression of *GA3ox1* and decreased expression of *GA2ox2* (Footitt *et al.*, 2011). Increased levels of *GA3ox* in response to stimuli of outgrowth have been reported in light-induced paradormant *Rosa* sp. buds (Choubane *et al.*, 2012), and in decapitated paradormant hybrid aspen buds (Rinne *et al.*, 2016).

The pattern observed in the transcript level of GA-responsive *VvGASA2* (Fig. 3), with highest levels during dormancy induction, a decrease towards dormancy maintenance, and no further changes during endodormancy release, is similar to the changes detected in the GA_1_ level. Assuming that *VvGASA2* transcription reflects the endogenous levels of GA (Herzog *et al.*, 1995; Aubert *et al.*, 1998), this similarity confirms the validity of the GA_1_ levels detected and their functional relevance. Of importance is the support for the absence of a significant increase in GA_1_ within the analyzed period (Fig. 2), in parallel with the increased GA biosynthesis ability and decreased GA inactivation ability during stages of dormancy release, as reflected by the transcript level.

The contradiction between the recorded increases in GA biosynthesis capacity—which supports the assumption that increased GA synthesis capacity is required either during or after endodormancy release—and the absence of accompanying increase in the GA level should be addressed. Here, we wish to note that while analyses under forcing conditions in growth chambers show that endodormancy is released, the bud population sampled in the vineyard for GA analysis is still ecodormant during January, and thus may not yet utilize its increased GA synthesis capacity. This may simply stem from the absence of a temperature appropriate for synthesis or may reflect the need for meristem activation for GA synthesis, as part of the regulated developmental program. In this regard, it is worth noting similar changes in levels of bioactive GAs recorded across the potato dormancy cycle in storage, using tubers that were transferred to forcing conditions (20^o^ C for 7 d) prior to GA analysis. In agreement with our finding, it was shown that (i) GA_4_ was not detected in potato buds; (ii) GA_1_ levels decreased during dormancy in storage; and (iii) GA_1_ levels increased only in tubers that had already exhibited actively elongating sprouts (Suttle, 2004).

The parallel decreases observed in *VvGA3ox* and *VvGA20ox* transcript levels and in the GA level following dormancy induction count against negative feedback regulation by GA, and suggest the involvement of another mode of regulation. The relatively high level of *VvGA2ox* may be a consequence of the high GA level at the beginning of the cycle. However, the absence of changes in its level during dormancy maintenance despite significant decreases in GA level question simple regulation, again suggesting contributions by other regulators. In both cases, ABA appears to be an appropriate candidate (Seo *et al.*, 2006; Toh *et al.*, 2008). In agreement with this, an increased synthetic capacity of ABA and an increase in its levels were previously recorded in grapevine buds during dormancy induction (Zheng *et al.*, 2015).

During dormancy release, the increases in *VvGA3ox* and *VvGA20ox* transcript levels and decreased *VvGA2ox* transcripts levels in principle agree with a GA feedback mechanism. Yet, some observations indicate that additional regulators may be involved in these changes: (i) a decreased GA level is evident from the end of November (the stage of dormancy maintenance, Fig. 2), but a sharp induction of *VvGA3ox2* and *VvGA20ox3* occurs only at the beginning of January; (ii) *VvGA20ox6*, which is also regulated by GA (Supplementary Fig. S1), is not induced in parallel with *VvGA3ox2* and *VvGA20ox3*; and (iii) expression levels of *VvGA2ox3* and *VvGA2ox4* significantly decrease at the onset of dormancy release, whereas the GA level remains stably low from the end of November. Again, in both cases, ABA is an appropriate candidate for regulation of these changes (Seo *et al.*, 2006). In agreement with this, increased degradation capacity of ABA and levels of its degradation products were previously recorded in grapevine buds during dormancy release (Zheng *et al.*, 2015).

### Exogenous GA delays bud dormancy release and limits the enhancing effect of HC

Based on the assumptions of the working model, and the above-described finding regarding changes in GA metabolism throughout the natural dormancy cycle, it was assumed that application of GA would bypass the need for regulated activation of GA biosynthesis during dormancy release, and serve as a direct stimulus of dormancy release. This assumption was supported by several reports in the literature: (i) GA_3_ application enhanced bursting of dormant Elberta peach buds (Donoho and Walker, 1957); and (ii) GA_4_ enhanced dormancy release of Japanese apricot flower buds (Zhuang *et al.*, 2013) and poplar buds (Rinne *et al.*, 2011).

The data presented in the current study do not support this prediction, as addition of exogenous GA_3_ did not stimulate grapevine bud break. On the contrary, a concentration-dependent inhibitory effect of GA_3_ was documented, both in the laboratory and in field trials (Fig. 4). Significant inhibition was also documented in response to GA_4 + 7_ (Supplementary Fig. S2). This inhibitory effect corroborates data reported in peach (Reinoso *et al.*, 2002), kiwi fruit (Lionakis and Schwabe, 1984), and persimmon (Kang *et al.*, 1998). A more complicated scenario has been described in poplar, where GA_3_ application led to bud abscission and protrusion of embryonic leaves, whereas GA_4_ induced bud burst (Rinne *et al.*, 2011). Indeed, it was previously reported that GA application prolonged grapevine dormancy (Weaver, 1959; Iwasaki, 1980). The delay exerted by exogenous GA on the advancing effect of HC (Fig. 5), which appeared to be concentration dependent as well, further suggests that GA somehow interferes with the cascade of biochemical changes required for bud break. Examples of a negative effect of GA on outgrowth of paradormant buds also appear in the literature, as described in the Introduction.

The changes recorded throughout the natural dormancy cycle and in response to exogenous GA treatments may seem contradictory at first glance. However, the contradiction may be reconciled by the hypothesis that the effects of GA treatment are a complex function that specifically depends on bud dormancy status. According to this hypothesis, increasing the level of GA by exogenous application prior to removal of the inhibition of meristem activity, an increase that is naturally inhibited in the buds at that stage, has a negative effect on dormancy release, whereas increasing its level after dormancy release has a positive effect on growth of bud primordia. This hypothesis is supported by the results of analysis of mature Arabidopsis seeds subjected to 7 d of imbibition in moist-chilled conditions, in which levels of GA_4_ (the biologically active GA detected in mature Arabidopsis seeds) decreased ~3-fold after 48 h, and increased only 18 h after transfer to germination conditions (Chiwocha *et al.*, 2005). The hypothesis may also be supported by the results of analysis of stored potato tubers, where (i) application of GA did not promote sprouting of deeply dormant tubers; (ii) exposure of tubers to inhibitors of GA biosynthesis did not extend tuber dormancy but rather hastened dormancy release; (ii) endogenous GA_1_ levels increased only in tubers that already exhibit actively elongating sprouts; and (iv) GA induces sprouting of non-dormant tubers (Suttle, 2004).

This hypothesis also serves to expand previous assumptions that an increase in endogenous GA may be a result rather than a cause of bud dormancy removal, and that GA may be needed for bud expansion after dormancy release (Saure, 1985; Lang, 1994).

### The negative effect of GA treatments on grapevine bud break depends on bud dormancy status

Treatment with GA had no negative effect on bud burst when applied 10 d after HC application (Fig. 7A). This suggests that: (i) the inhibitory effect of exogenous GA is not a non-specific, wide-ranging suppressive effect on bud primordial growth activity; and (ii) the inhibitory effect of GA on HC-treated buds is greatest when GA is applied either when the meristem activity is still repressed or during the very early stages of removal of repression. However, when applied after dormancy is released, GA would not have an inhibitory effect. In that regard, it is instructive to note that HC-induced biochemical changes start as early as 24 h after treatment, level off at 96 h, and lead to actual burst after 14 d or earlier, depending on dormancy status (Ophir *et al.*, 2009). Hence, at 10 d, the bud meristem activity may already have resumed.

The inhibitory effect of GA, when applied to pre-chilled buds or to naturally ecodormant buds in parallel with forcing initiation, and its enhancing effect when applied 7 d and 3 d later, respectively (Fig. 7B, C), further suggest that GA application during initial activation of the endo- or ecodormant woody bud meristem has a negative effect on meristem activation, while its application to an already activated meristem enhances regrowth.

Support for this idea comes from (i) the inhibitory effect of GA_3_ on kiwi fruit bud break when applied before chilling accumulation, and its promoting effect when applied after exposure to optimal chilling hours which enhance dormancy release (Lionakis and Schwabe, 1984); and (ii) the observation that application of biologically active GAs was markedly less effective than application of fluridone, an inhibitor of ABA synthesis, for enhancement of dormancy release of Arabidopsis seeds. Interestingly, combined application of fluridone with GA_3_ improved the enhancement of dormancy release, compared with that of fluridone alone. It was therefore suggested that a decrease in ABA level and dormancy intensity may be a prerequisite for a positive effect of elevated levels of GA and sensitivity to GA during seed germination (Ali-Rachedi *et al.*, 2004).

### The expression profiles of GA metabolism regulators in response to artificial stimuli of bud break suggest complex and dormancy status-dependent regulation

The improved bud break of combined HC–GA-treated buds, as compared with buds treated with GA alone (Fig. 5A, B), suggests that exposure to HC may facilitate manipulation of the artificially increased levels of GA. In a wider view, HC may enhance bud break through precise regulation, over time, of the levels of bioactive GA in the buds: preventing a rise of GA levels in the buds prior to meristem growth, whilst enhancing GA biosynthesis at or after resumption of meristem growth.

In line with the above, HC application resulted in decreases and increases, respectively, of bud-expressed, functionally characterized GA biosynthesis and deactivation genes (Giacomelli *et al.*, 2013) 12–24 h following HC application (Fig. 6A, C–E). Interestingly, a similar scenario was recently described during natural dormancy release of Japanese pear flower buds (Bai *et al.*, 2013). This provides support for the suggestion that temporary limitation of GA availability is particularly important at the time of removal of the dormancy block. The significant decrease in GA level at 48 h after HC application (Fig. 5C) supports the idea that the effect of HC on transcription indeed leads to a temporary decrease in GA availability. The decreased expression of the GA-responsive *VvGASA2* 24–48 h after application of HC (Fig. 6F) further supports the suggestion that treatment with HC reduces the GA level within this time window.

The effect of HC was multifaceted and dependent on time of application, as a reverse effect was evident at 96 h (i.e. an increased transcript level of GA biosynthesis enzymes and *VvGASA2*, and a decrease of GA deactivation enzymes, Fig 6A, C–F). These opposing effects of HC, which depend on dormancy status (assuming that at 96 h repression was removed), suggest that the effect of HC over time may involve optimized co-ordination of hormonal interactions during the cascade of events that start with dormancy release and continue to meristem activation. According to the suggested scenario, HC initially ‘guarantees’ that GA availability is limited until dormancy is released, and later increases in GA availability enhance primordial regrowth. The validity of the timing-dependent reverse effects of HC on expression of GA biosynthesis and catabolism regulators is supported by similar effects recorded in response to other dormancy release stimuli, such as hypoxia and HS (Fig. 6G–K, M–Q). It is also supported by the expression profiles of *VvGASA2* (Fig. 6L, R). The decreased GA level observed in Arabidopsis seeds during imbibition, and its increase only after transfer to conditions that favor germination, also agrees with the hypothesis (Chiwocha *et al.*, 2005).

### Does the GA inhibitory effect operate via inhibition of CK function during meristem activation?

The results that have been presented clearly demonstrate an inhibitory effect of GA when applied to dormant grapevine buds, and there are indications for its relevance in natural situations. However, the detailed nature of this inhibitory effect is not yet clear.

Interestingly, GA is known to repress numerous CK responses in various developmental processes in different plants (Greenboim-Wainberg *et al.*, 2005; Fleishon *et al.*, 2011). Among various antagonistic effects, it was shown that GA constitutive signaling has a detrimental effect on shoot apical meristem (SAM) function, which requires CK for establishment and maintenance of meristematic identity (Jasinski *et al.*, 2005; Skylar and Wu, 2011, and references within). A positive role for CK in regulation of paradormant bud outgrowth is also well established (Domagalska and Leyser, 2011; Müller and Leyser, 2011; Braun *et al.*, 2012; Dun *et al.*, 2012). In light of the above, we propose a speculative scenario in which the GA inhibitory effect on grapevine bud break results from its antagonistic effect on CK responses required for activation of the bud meristem. In agreement with this, decreased CK levels in potato tubers by overexpression of *cytokinin oxidase/dehydrogenase1* (*CKX*) resulted in a prolonged dormancy period which was not affected by GA_3_. It was therefore suggested that CK has an essential role in terminating tuber dormancy, whereas GA supports sprout growth after dormant meristem activation (Hartmann *et al.*, 2011). Interestingly, preliminary analysis revealed that CK levels are dramatically increased during natural and artificial dormancy release, supporting the assumption that CK plays a role during grape woody bud meristem activation. The suggested scenario, and its underlying assumptions, will require future detailed study.

In light of the above, a speculative working model is suggested, as a foundation for further study. According to this model, the GA level decreases during dormancy induction, due to down-regulated expression of GA biosynthesis genes. A parallel increase of ABA biosynthesis capacity may be involved in regulation of such decreased GA biosynthetic capacity. GA is then maintained at a low level until the meristem is activated, since its premature increase will inhibit meristem activation. Inhibition may be mediated by repression of CK responses which are involved in regulation of meristem activation. Once the meristem is activated, GA is required to support primordial growth and bud burst. Increased ABA degradation capacity during dormancy release may participate in up-regulation of GA biosynthetic capacity, by induction of *GA3ox* and *GA20ox* expression. Low levels of GA during dormancy release may also contribute to such up-regulation of expression by a negative feedback mechanism.

## Supplementary data

Supplementary data are available at *JXB* online.

Table S1. Schematic details of all the GA treatments.

Table S2. Primers used for gene expression analyses by qRT-PCR.

Fig. S1. The effect of GA_3_ application on the expression profiles of the GA-responsive *VvGASA2* gene and the central components of GA metabolism.

Fig. S2. The effect of GA_4 + 7_ application on bud break of single-node cuttings.

Supplemental Figures and Tables
