# Supplementary material for: Distinct gibberellin functions during and after grapevine bud dormancy release
Source: J Exp Bot. 2018 Jan 27;69(7):1635–48. doi: 10.1093/jxb/ery022 (PMC5888973; doi:10.1093/jxb/ery022)
Supplement: Supplemental Figures and Tables [file ery022_suppl_supplemental_figures_and_tables.pdf]

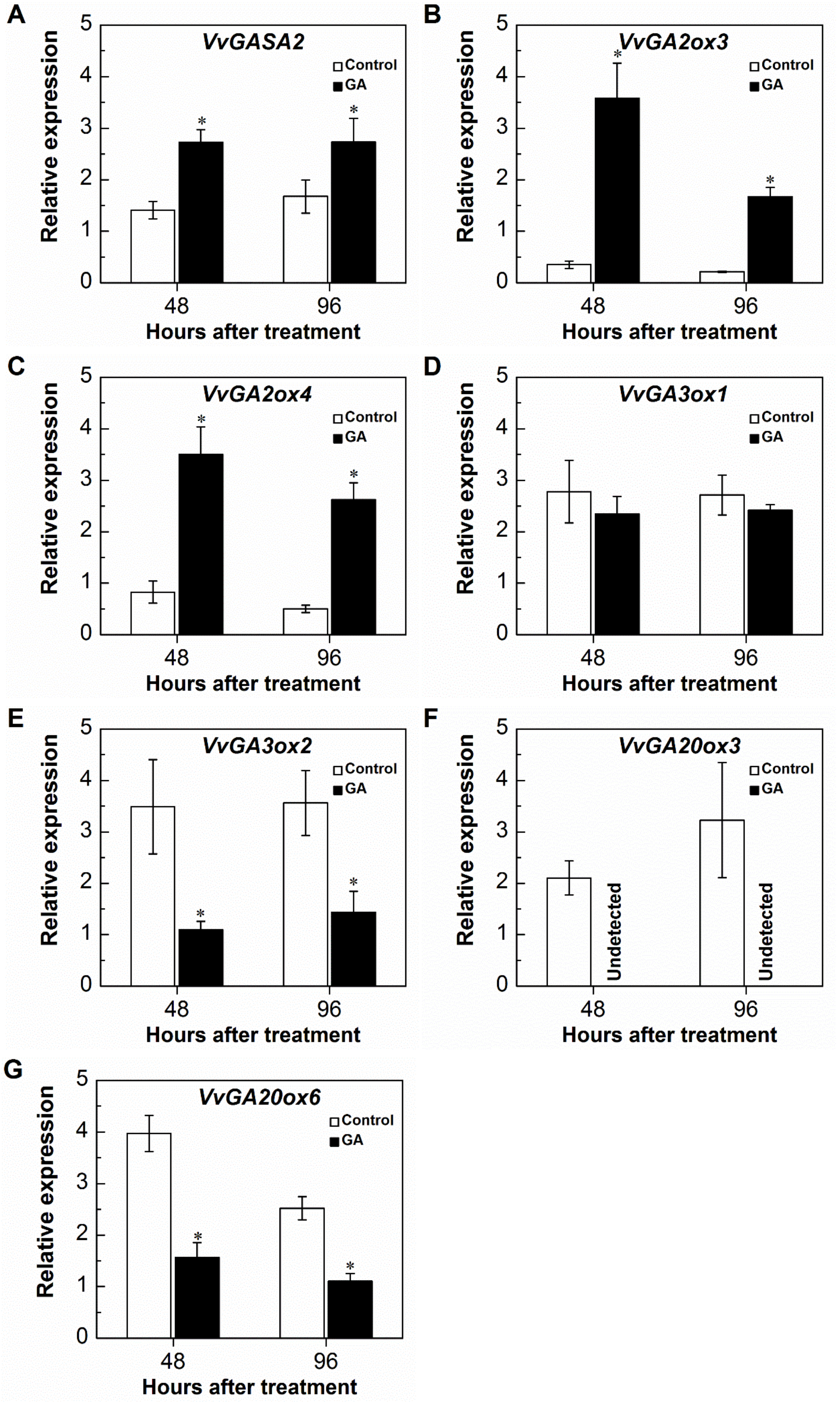

Figure S1. The effect of GA_3 application on the expression profiles of the GA responsive *VvGASA2* gene and the central components of GA metabolism. Total RNA was extracted from control (0.02% Triton X-100) and GA (10 ppm GA_3 with 0.02% Triton X-100) at 48 and 96 h after treatment, as described in Materials and Methods. Relative expression of *VvGASA2* (**A**), *VvGA2ox3* (**B**), *VvGA2ox4* (**C**), *VvGA3ox1* (**D**), *VvGA3ox2* (**E**), *VvGA20ox3* (**F**) and *VvGA20ox6* (**G**) were determined by qRT-PCR, as described in Materials and Methods, normalized against *VvActin* and *VvGAPDH*. Values represent the mean expression ± SE of three biological replications, each with two technical repeats. Asterisks between treatments indicate significantly differences according to Student's t-test (*, $P < 0.05$).

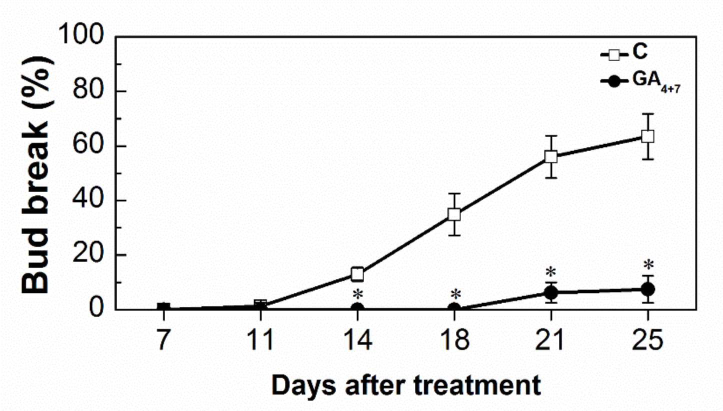

Figure S2. The effect of GA_{4+7} application on bud break of single node cuttings. Cuttings were sprayed with 10 ppm GA_{4+7} (ratio of GA_4 to GA_7 is 2:1, Duchefa, Haarlem, The Netherlands) formulated with 0.02% Triton X-100, placed in vases and monitored for 25 d as described in Materials and Methods. Triton X-100 (0.02%)-treated buds served as control. Values are averages of nine groups of replications, consisting of 10 buds each ± SE. Asterisks between treatments indicate significantly differences according to Student's t-test (*, $P < 0.05$).

Table S1: Schematic details of all the GA treatments

Figure	GA type	GA app day	GA (ppm)	3% HC app day	Treatment label	Monitoring days from 0 d
4A	GA_3	none	none	none	**Control**	24, 28
4A	GA_3	0 d	1.25	none	**GA (1.25)**	24, 28, 67
4A	GA_3	0 d	2.5	none	**GA (2.5)**	24, 28, 67
4A	GA_3	0 d	5	none	**GA (5)**	24, 28, 67, 77, 87, 95
4A	GA_3	0 d	10	none	**GA (10)**	24, 28, 67, 77, 87, 95, 107
4A	GA_3	0 d	20	none	**GA (20)**	24, 28, 67, 77, 87, 95, 107
4A	GA_3	0 d	40	none	**GA (40)**	24, 28, 67, 77, 87, 95, 107
4B	GA_3	none	none	none	**Control**	7, 10, 14, 17, 21, 24, 28
4B	GA_3	0 d	0.001	none	**GA (0.001)**	7, 10, 14, 17, 21, 24, 28
4B	GA_3	0 d	0.01	none	**GA (0.01)**	7, 10, 14, 17, 21, 24, 28
4B	GA_3	0 d	0.1	none	**GA (0.1)**	7, 10, 14, 17, 21, 24, 28
4B	GA_3	0 d	1	none	**GA (1)**	7, 10, 14, 17, 21, 24, 28
S1	GA_{4+7}	none	none	none	**Control**	7, 11, 14, 18, 21, 25
S1	GA_{4+7}	0 d	10	none	**GA_{4+7}**	7, 11, 14, 18, 21, 25
4C	GA_3	none	none	none	**Control**	40, 48, 55
4C	GA_3	0 d	10	none	**GA**	40, 48, 55
5A	GA_3	none	none	none	**Control**	11, 14, 18, 21, 25
5A	GA_3	0 d	1	none	**GA (1)**	11, 14, 18, 21, 25
5A	GA_3	0 d	5	none	**GA (5)**	11, 14, 18, 21, 25
5A	GA_3	0 d	10	none	**GA (10)**	11, 14, 18, 21, 25
5B	GA_3	none	none	0 d	**HC**	11, 14, 18, 21, 25
5B	GA_3	0 d	1	0 d	**HC-GA (1)**	11, 14, 18, 21, 25
5B	GA_3	0 d	5	0 d	**HC-GA (5)**	11, 14, 18, 21, 25
5B	GA_3	0 d	10	0 d	**HC-GA (10)**	11, 14, 18, 21, 25
7A	GA_3	none	none	none	**Control**	11, 14, 18, 21
7A	GA_3	none	none	0 d	**HC**	11, 14, 18, 21
7A	GA_3	0 d	10	0 d	**HC-GA (0 d)**	11, 14, 18, 21
7A	GA_3	2 d	10	0 d	**HC-GA (2 d)**	11, 14, 18, 21
7A	GA_3	6 d	10	0 d	**HC-GA (6 d)**	11, 14, 18, 21
7A	GA_3	10	10	0 d	**HC-GA (10 d)**	11, 14, 18, 21
7B	GA_3	none	none	none	**Control**	7, 10, 11, 13, 14, 17, 21
7B	GA_3	0 d	1	none	**GA (0 d)**	7, 10, 11, 13, 14, 17, 21
7B	GA_3	7 d	1	none	**GA (7 d)**	7, 10, 11, 13, 14, 17, 21
7C	GA_3	none	none	none	**Control**	3, 4, 5, 6, 7, 10, 14
7C	GA_3	0 d	1	none	**GA (0 d)**	3, 4, 5, 6, 7, 10, 14
7C	GA_3	3 d	1	none	**GA (3 d)**	3, 4, 5, 6, 7, 10, 14

Table S2: Primers used for gene expression analyses by qRT-PCR

	**Forward primer (5' to 3')**	**Reverse primer (5' to 3')**	**Accession number**
VvGA3ox1	CCTCAGAACTCGTGGGTCAT	TGGGCAGGTAGAGAGAAAAGG	VIT_09s0002g05270[a]
VvGA3ox2	CCCCACATACAGACTCATTGC	TCCACCCAACTCCATCTCTC	VIT_04s0008g04940[a]
VvGA20ox3	GCCTAAAACCCGACCTCACT	GGACCACCATTTGTCATCTACA	VIT_16s0022g02310[a]
VvGA20ox6	GGTGTTCTTCGTGTGCCCTA	CTTCCTTGTCCCTTCTCTGC	VIT_18s0001g01390[a]
VvGA2ox3	TGTGAAGCATAGGGTGTTGA	ATGAGTGAGGGCAATGGTG	VIT_19s0140g00120[a]
VvGA2ox4	GCTCTGCTGTGAGTGGTTACA	ATGTGTGATGAGGCTGCTGA	VIT_05s0077g00520[a]
VvGASA2	CCCAAACCCCTCTCCTCTTTC	GGCGATCCAATGCTTCGAAC	VIT_14s0108g00740[a]
VvGAPDH	TTCTCGTTGAGGGCTATTCCA	CCACAGACTTCATCGGTGACA	CB973647[b]
VvActin	CTTGCATCCCTCAGCACCTT	TCCTGTGGACAATGGATGGA	EC969944[b]
[a]Accessions from Ensemblplants: http://plants.ensembl.org/index.html			
[b]NCBI accessions			
